# Morphological Variability and Clinical Significance of the Fibularis Tertius Muscle: An Extensive Literature Review

**DOI:** 10.3390/jcm14113991

**Published:** 2025-06-05

**Authors:** Marta Pośnik, Andrzej Węgiel, Nicol Zielinska, Kacper Ruzik, Łukasz Olewnik, George Triantafyllou, Maria Piagkou, Michał Podgórski

**Affiliations:** 1 Department of Radiology, Diagnostic Imaging and Interventional Radiology, Medical University of Lodz, 90-419 Lodz, Poland; marta.posnik@stud.umed.lodz.pl (M.P.); michal.podgorski@umed.lodz.pl (M.P.); 2 Department of Rheumatology, Medical University of Lodz, 90-549 Lodz, Poland; andrzej.wegiel@stud.umed.lodz.pl; 3 Department of Clinical Anatomy, Masovian Academy in Płock, 09-402 Płock, Poland; n.zielinska@mazowiecka.edu.pl (N.Z.); k.ruzik@mazowiecka.edu.pl (K.R.); 4 Department of Anatomy, School of Medicine, National and Kapodistrian University of Athens, 11527 Athens, Greece; georgerose406@gmail.com (G.T.); mapian@med.uoa.gr (M.P.)

**Keywords:** fibularis tertius, peroneus tertius, anterior compartment, leg, variation

## Abstract

**Background:** The muscles and their tendons exhibit considerable morphological variations. While the anterior leg compartment may seem uniform, several well-documented variants of the tibialis anterior, extensor hallucis longus (EHL) and extensor digitorum longus (EDL) exist. In contrast, little is known about the fibularis tertius muscle (FT). This literature review aims to compile existing data on the FT and its variations and assess this structure’s clinical significance. **Material and Methods:** This comprehensive literature review is based on scientific articles obtained from PubMed. All relevant papers were included, and citation tracking was conducted to ensure a thorough examination of the topic. **Results:** This detailed literature review synthesizes the latest scientific findings regarding the FT, exploring its variable morphology, functional anatomy, evolutionary significance and clinical relevance. A high morphological variability of the FT is described including its origin, insertion and accessory form. Nevertheless, the FT has been described in cadaveric studies between adults and fetuses, while few classification systems have been proposed. **Conclusions:** The FT is an intriguing structure that has garnered interest from researchers across various fields, including medicine, clinical practice and biological sciences. There are few clinical implications of the muscle such as FT syndrome or tendon tear. Adequate knowledge of its anatomy is of paramount importance for clinicians.

## 1. Introduction

Along with the tibialis anterior muscle (TA), extensor hallucis longus (EHL), extensor digitorum longus (EDL) and the fibularis or peroneus tertius (FT or PT) muscles form the anterior leg compartment. Among all four components, the FT is positioned as the most superficial layer [1].

The FT is typically described as a structure originating from the distal third or half of the fibula and the intermuscular septum. It travels caudally and laterally, transforms into the fibularis tertius tendon (FTT) and then inserts into the base of the fifth metatarsal bone [1]. Its most well-known function is to support the lateral longitudinal arch, allowing for equal pressure distribution. Numerous variants among the described muscular components have been noted and thoughtfully examined, including additional tendons of the EHL and the depiction of TA tendon (TAT) anatomy [2,3]. When reviewing the literature on this compartment, many issues related to FT morphology and its clinical significance still need clarification.

The present literature review sought to systematically organize the existing data on FT and evaluate its reported variants, evolutionary development, imaging and clinical implications. Furthermore, we aimed to identify the limitations of the published literature and recommend areas that warrant further investigation.

## 2. Methods and Review Design

An online search was conducted in the PubMed database to locate appropriate publications for the literature review. The following terms were used during the search strategies: fibularis tertius, peroneus tertius, peroneus tertius function, fibularis tertius function, fibularis tertius variation, peroneus tertius variation, anterior compartment of the leg anatomy, anterior compartment of the leg variation, fibularis muscles, peroneus muscles, fibularis muscles variation, peroneus muscles variations. The exclusion criteria were as follows:

- Manuscripts written in any language other than English.
- Insufficient information regarding the description of the study.
- Types of articles that include expert opinions, letters to the editor or conference reports.
- Publications dated after August 2024.

We extracted valuable information concerning the subject matter from each article selected for review, organizing it according to each section and subsection for the literature review. This manuscript included 47 papers, and citation tracking was conducted using Mendeley Reference Manager (version 2.114.0) to identify the sources. The review is structured into the following sections: classical description and function-reported variations among adults, embryological development, visualization and imaging studies, and clinical applications. Each section is further divided into subsections to enhance the information’s clarity and understanding.

## 3. Results and Discussion

### 3.1. The Fibularis Tertius (FT) Typical Descriptive and Functional Anatomy

#### 3.1.1. Typical Descriptive Anatomy

**Figure 1:**
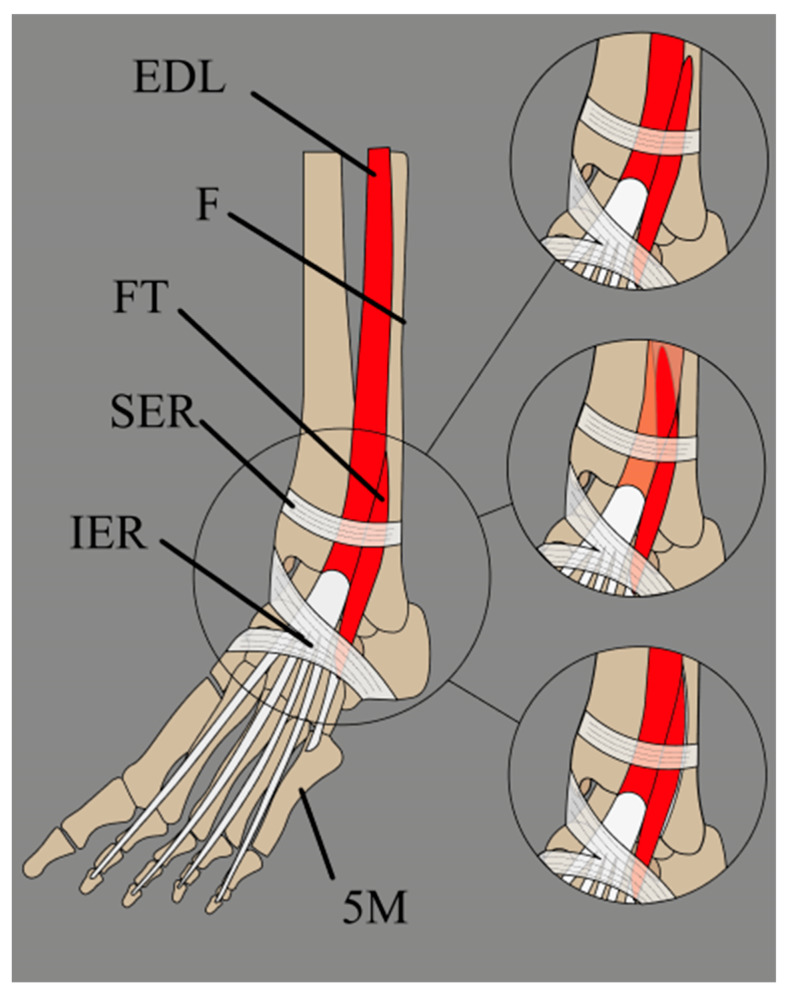
Schematic presentation of the fibularis tertius muscle, with its three most common insertion sites. EDL: extensor digitorum longus muscle; F: fibula; FT: fibularis tertius; SER: superior extensor retinaculum; IER: inferior extensor retinaculum; 5M: fifth metatarsal bone.

In scientific literature and anatomy textbooks, the FT is commonly referred to by one of three names: fibularis tertius, peroneus tertius or anterior fibularis. The FT is the most superficial muscle in the anterior leg compartment [1,4]. According to classical anatomy descriptions, it typically appears as a direct continuation of the EDL. It originates from several locations, including the distal third of the fibula’s medial surface, the interosseous membrane’s anterior surface and/or the anterior intermuscular septum [1,4]. The muscle belly descends in an inferolateral direction, forming a fibrous tendon that travels beneath the superior extensor retinaculum (ER) and continues towards the inferior ER, where it is accompanied by the EDL, ultimately attaching to the fifth metatarsal bone [1,4] (Figure 1).

#### 3.1.2. Functional Anatomy

The FT’s primary role is to support the lateral longitudinal arch of the foot, ensuring an even distribution of pressure. Jungers et al. [5] conducted an interesting electromyographic study that found that the FT is active only during bipedalism’s gait phase, classifying it as a secondary gait muscle [5]. The fibularis longus and brevis muscles (FL and FB) assist this movement. Consequently, if the FT is absent or damaged, the FL and FB must compensate for the eversion of the foot. The contraction of all the muscles in the anterior compartment produces dorsiflexion, which raises the foot to a flat position. The primary purpose of the FT movement is to reposition the toes before the next support phase, helping to prevent tripping and enhancing movement efficiency [5]. Rourke et al. [6] suggested that the FT extends the ankle and flexes the subtalar joints. According to Yammine and Eric [7] FT facilitates strong foot eversion. A study by Witvrouw et al. [8] found that the absence of the FT does not reduce movement strength in eversion, dorsiflexion or other motions. While the absence of the FT does not affect strength during these movements, electromyographic studies indicate that it plays a vital role during the walking phase. This is further supported by the observation that other muscles compensate for the FT function when absent.

### 3.2. Reported Variants of the Fibularis Tertius (FT)

#### 3.2.1. Prevalence of the FT Presence

According to various studies, the prevalence of FT in adults ranges from 88.2% to 100%. A meta-analysis conducted by Yammine and Erić [7] found that the FT is highly prevalent in humans, with a presence in 93% of cases. This prevalence of 93% is higher than that reported in some other reviews and texts. The frequency of FT varies significantly among different populations. For instance, Kimura and Takahashi [9] reported a prevalence of 4.8%. The FT is most frequently observed in South American and Japanese populations, with prevalence rates of 97.4% and 95.5%, respectively. Conversely, populations such as Africans, Indians and Chinese exhibit lower incidence rates at 90.2%, 90.8% and 89.3%, respectively (Table 1). Yammine and Erić suggest that these differences may be attributed to genetic factors influencing the presence of the FT. Romanes indicated that the FT is often absent, but according to the study by Yammine and Erić, an absence of the FT is relatively rare [10].

Interestingly, no differences were found regarding side prevalence. Yammine and Erić reported a mild association favoring males, while Palomo-Lopez et al. [11] found no association between sex and FT prevalence.

Eliot et al. [12] conducted a study investigating whether the fifth metatarsal bone’s morphology affects the FT’s presence. The study evaluated two criteria: the dorsal shaft edge’s sharpness and the dorsal tubercle’s size and prominence. The authors concluded that the morphology of the fifth metatarsal does not predict the presence or absence of the FT.

It is important to note that the presence of the FT varied between cadaveric and clinical studies. Cadaveric dissections showed a higher frequency (93%), while clinical examinations revealed a lower frequency (80%). This discrepancy may be explained by the difficulty separating the FT tendon (FTT) and/or FT belly from the EDL during clinical assessments. This challenge can lead to a lower observed frequency in clinical settings, as palpation may not effectively distinguish the FT from the EDL lateral slip.

#### 3.2.2. Variations in the Origin of the Fibularis Tertius (FT)

**Figure 2:**
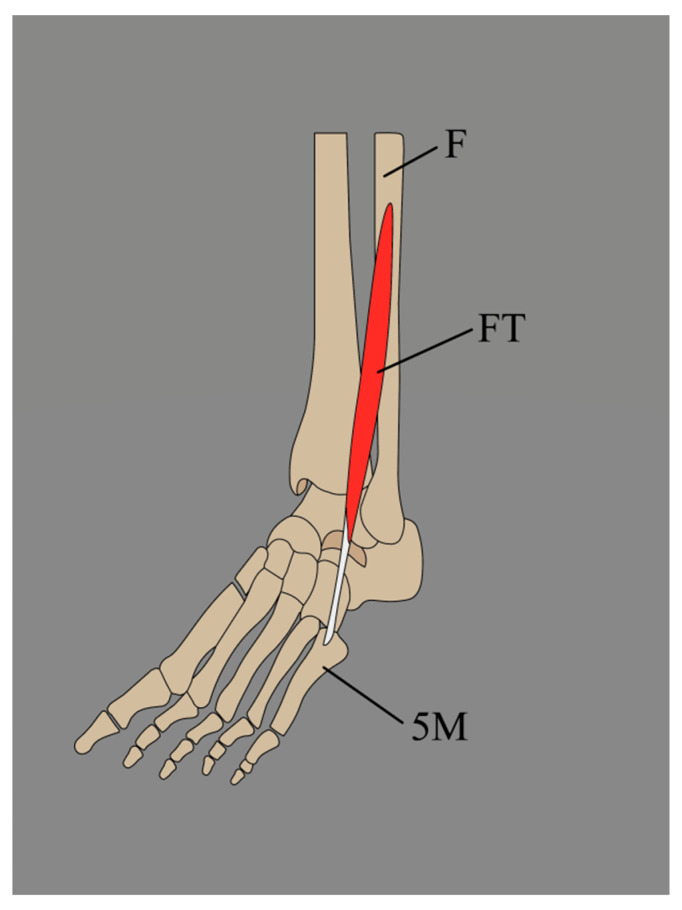
Fibularis tertius presented by Yammine and Erić. F: fibula; FT: fibularis tertius; 5M: fifth metatarsal bone.

According to classical anatomical description, the FT originates from the distal third of the fibula as a direct continuation of the attachment of EDL from the interosseus membrane and/or anterior intermuscular septum. However, Yammine and Erić [7] determined that the FT originates from the distal half rather than the distal third or fourth of the fibula (Figure 2, Table 2).

#### 3.2.3. Variations in the Insertion of the Fibularis Tertius (FT)

FT’s insertion presents significantly greater variation compared to its origin. Numerous reports have documented cases of a doubled FTT [3,13,14] Several classifications of the FT insertion have been proposed (Table 3). Among these, Olewnik et al. put forth the most recent classification [15], which identifies six distinct types described as follows:

- A single tendon, band-shaped, inserted into the shaft of the fifth metatarsal bone (45%).
- A single tendon, fan-shaped, inserted at the base of the fifth metatarsal bone (22%).
- A single tendon, fan-shaped, inserted into the fascia covering the fourth interosseous space and the base and shaft of the sixth metatarsal bone (16.5%).
- A bifurcated tendon consisting of both band and fan shapes. The main fan-shaped tendon is inserted at the base of the fifth metatarsal bone, while the accessory tendon is attached to its shaft (8.8%).
- A bifurcated tendon, fan-shaped, where the main tendon is inserted at the base of the fifth metatarsal bone, and the secondary tendon attaches to the base of the fourth metatarsal bone (5.5%).
- This tendon fuses with an additional band from the FB, which gives rise to the fourth interosseous dorsalis muscle (2.2%).

Interestingly, older anatomical reports also mention trifurcated FTTs. Souligoux dissected an FT whose trifurcated tendon (FTT) was inserted on the third, fourth and fifth metatarsals and the corresponding interosseus spaces [16]. In a report by Cuyer, at the distal third of the leg, the FT’s muscle belly is divided into three bundles, each supplying a tendon. The most lateral tendon is inserted at the base of the fifth metatarsal bone, and the second tendon, medial to the previous one and equal in size, is inserted in the superior border of the shaft of the fifth metatarsal bone. The third tendon, the most medial one, is inserted in the two distal phalanges of the fifth toe [16].

#### 3.2.4. Fibularis Tertius Tendon (FTT) Morphology

**Figure 3:**
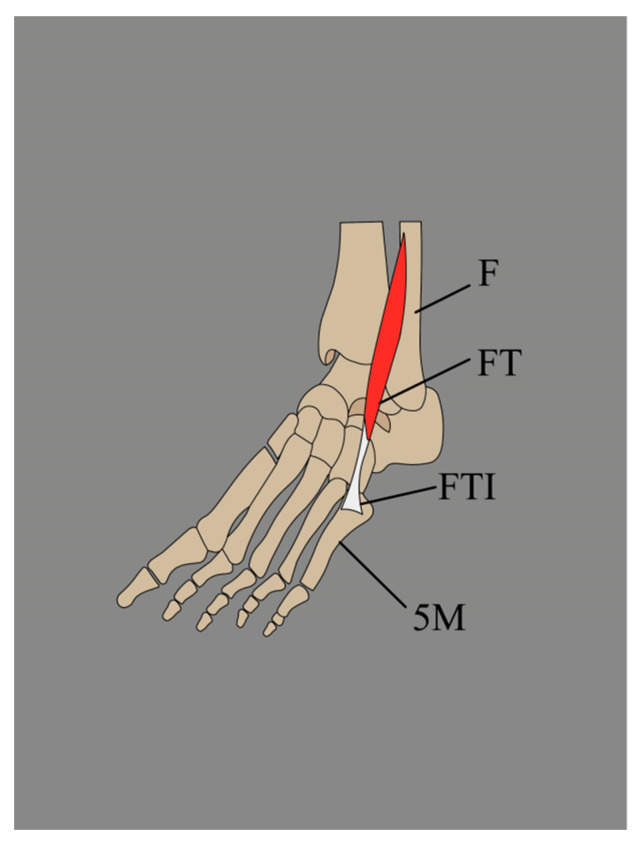
Band-shaped insertion of the fibularis tertius. F: fibula; FT: fibularis tertius; FTI: fibularis tertius insertion; 5M: fifth metatarsal bone.

According to Olewnik et al. [17,18], there are two possible types of FTT insertions: band- and fan-shaped. Band-shaped insertion is less than twice the width of the tendon above (Figure 3). In contrast, fan-shaped insertion is characterized as an insertion at least twice the width of the tendon above. In their publication that assessed FTT morphology, Olewnik et al. [15] observed the band-shaped variant in conjunction with Type I insertion of the FTT. In Type IV, the accessory band connected to the shaft of the fifth metatarsal bone was also band-shaped. The fan-shaped variant was found with Type II, III, IV and V insertions of the FTT. In Type V, the accessory band that attached to the base of the fourth metatarsal bone was also fan-shaped. Variants in Type VI were not evaluated since the FTT was fused with the EDL tendon (EDLT) [15].

#### 3.2.5. FT Accessory Forms

Interestingly, duplication of the entire FT is exceedingly rare; however, it has been reported bilaterally. According to Bejjani et al. [16] Le Double found a complete, bilateral duplication in 6 cadavers. An accessory muscle (AFT) associated with the FT is also called fibularis quinti digiti. This accessory muscle, usually, if present, arises from the tendon of the FB proximal to the lateral malleolus, at approximately the level of the superior fibular retinaculum [19]. However, in rare cases, when the EDL lacs a tendon to the fifth toe, it might be replaced by the fibularis quinti digiti that originates from the common muscle belly with the FT to insert into the extensor expansion of the fifth toe [3]. As mentioned in Bejjani et al. [16] transcript, the peroneal muscles derive from the same muscle mass as the extensors of the toe; therefore, the variability of the fibularis quinti digit’s origin seems justified.

## 4. Embryological Development and Occurrence Among Other Species

### 4.1. Evolution and Prevalence Among Other Species

For many years, the FT was considered a trait unique to humans; however, this assumption has been definitively disproven. There are two reports documenting the FT’s occurrence among Old World monkeys: the pig-tailed baboon (*Papio ursinus*), the toque monkey (*Macaca sinica*) and crab-eating monkeys (*Macaca fascicularis*) [7,9,20]. In great apes, Loth et al. reported FT’s prevalence as 6.6% among orangutans and stated 5% occurrence among chimpanzees [21]. Kimura et al. [9] presented a 29.6% prevalence *in gorillas*. Such findings suggest that FT’s presence is highly connected with bipedalism due to its predominant presence in humans and gorillas, which are exclusively and primarily terrestrial. Such a hypothesis might also be applied to chimpanzees—lesser FT’s presence might relate to the fact that they are not fully terrestrial but both terrestrial and arboreal [7,22]. Interestingly, foot inversion in dorsiflexion is not counteracted by any muscle in arboreal apes, and FT’s eversion function was stated as a characteristic feature of human locomotion [7]. Jungers [5] named it a swing phase acting to level the sole before the next touchdown. It has been observed that when the lateral border of the foot is elevated and pronated, the action of the FT shifts the weight distribution toward the medial arch, helping to maintain balance during the stance phase [5,23]. Therefore, FT could help improve the economy of bipedal walking.

### 4.2. Embryological Development Among Humans

The embryological development of the FT is a subject of controversy. According to the most common description, in the early developmental stages, the common extensor and peroneal mass are connected [13]. In a 14 mm embryo, those masses diverge. The extensor mass is differentiated into the TA, EDL and EHL [24]. The FT is early distinct from the EDL. According to Baarden [25] the muscle appears in the crown-rump length of the fetus, which is around 20 mm. Nonetheless, some authors state that the FT might derive from the short, deep muscles of the extensor leg compartment. A 3D immunostaining embryological study conducted by Diogo et al. might support this thesis [26]. The authors found an interesting configuration in a 46 mm embryo, where the part of the extensor digitorum brevis (EDB) that inserts to the fourth digit was continuous with a fragile muscle structure located fibular to it and that, in terms of its distal portion, was located above the region of the fifth metatarsal—where the FT of adults is typically positioned. Interestingly, the authors did not find such a structure during an examination of the 51 mm embryo [26] it cannot be ruled out whether the observed thin structure was a case of an anatomical variation seen in a single fetus or if it is a relatively constant structure. Further embryological studies are needed to understand FT’s development fully.

### 4.3. Prevalence of the FT Among Fetuses

FT’s prevalence among fetuses varies between studies. In a meta-analysis by Yammine et al. [7] published in 2017, the mean prevalence was 82.1%. Since then, three more studies have been published. Albay and Candan [27] reported that the muscle was present in 80% of cases (160 out of 200 limbs). In the second one by Karauda et al. [24] FT was present in 50% of cases (out of 100 limbs examined, 50 presented FT). Results from the Ruzik et al. [28] study corresponded with the results presented by Karauda et al. [24] (Table 4).

### 4.4. Variation of the FT Origin Among Fetuses

The origin of the FT is often described as a consistent structure. Ruzik et al. [28] conducted a study involving 100 lower limbs from 50 spontaneously aborted human fetuses, ranging from 18 to 38 weeks of gestation. The authors identified four distinct types of FT origin:

I—Origin, located at the proximal third of the fibula and the intermuscular septum (5 cases).

II—Origin, located at the middle third of the fibula and the intermuscular septum (21 cases).

III—Absent FT muscle belly and the independent FTT originates from the extensor digitorum longus tendon (EDLT) (8 cases).

IV—Origin, located at the distal third of the fibula and the intermuscular septum (16 cases).

### 4.5. Variation of the FT Insertion Among Fetuses

Karauda et al. [24] conducted a study on the insertion of the FT among 50 spontaneously aborted fetuses. They examined 100 limbs and identified FT in 50 of those. The study distinguished the following types of FT insertions:

I—Single distal attachment: the FTT inserted onto the shaft of the fifth metatarsal bone (9 cases).

II—Single distal attachment: The FTT is characterized by a very broad insertion at the base of the fifth metatarsal bone (2 cases).

III—Single distal attachment: the FTT inserted onto the shaft of the fourth metatarsal bone and the fascia covering the fourth interosseous space (6 cases).

IV—Single distal attachment: the FTT inserted into the fascia covering the fourth interosseous space (10 cases).

V—Bifurcated distal attachment: the main tendon has a very broad insertion at the base of the fifth metatarsal bone, while the accessory band inserts into the base of the fourth metatarsal bone (7 cases).

**Figure 4:**
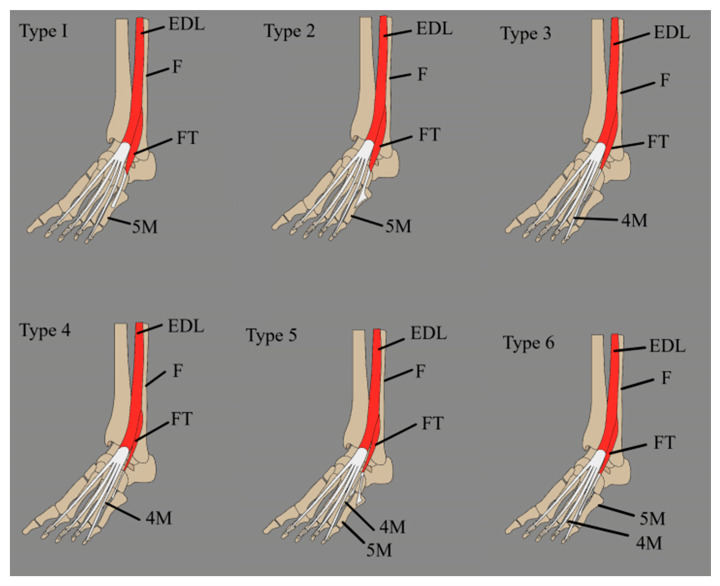
Fibularis tertius classification by Karauda et al. [24]. EDL: extensor digitorum longus; F: fibula; FT: fibularis tertius; 4M: fourth metatarsal bone; 5M: fifth metatarsal bone.

VI—Bifurcated distal attachment: the main tendon inserts into the base of the fourth metatarsal bone, and the accessory bands insert into the fourth interosseous space (16 cases) (see Figure 4).

### 4.6. Fibularis Tertius Tendon (FTT) Morphology in Fetuses

According to Karauda et al. [24], the most common tendon morphology observed in fetuses is the fan-shaped type, which can be classified into types I, II, III and V. In contrast, the band-shaped variant is rare and is mainly found in types V and VI.

### 4.7. Accessory Forms of FT in Fetuses

Albay and Candan [26] presented a study that explores the clinical significance of fibular muscles and assesses the prevalence of FTs. The authors surveyed 200 limbs from 100 embalmed fetuses, revealing interesting results. They found that accessory fibular muscles were present in 3.5% of the dissected limbs, while FT was absent in 20% of cases. Furthermore, the authors classified the AFT into two types: Type A, known as fibularis quartus, which derives from the FB, and Type B, called fibulocalcaneus externus, which originates from the distal part of the fibula. It can be inferred that in the absence of FT, AFT may assist in performing some of the lost functions, such as stabilizing the ankle joint during movement. However, further research on this topic is necessary.

## 5. Visualization and Imaging Studies

Magnetic resonance imaging (MRI) is widely recognized as the standard method for assessing the structure and condition of soft tissues. It is particularly effective in identifying and evaluating the extent of injuries such as muscle tears, oedema, fat atrophy and other pathological changes. MRI allows for detailed visualization of structures and provides high contrast between different types of tissues, making it an essential tool for diagnosing complex soft tissue conditions. MRI is consistently identified as the preferred imaging modality in the literature concerning foot and ankle injuries. It offers precise insights into the structural integrity and functional status of muscles. Its versatility and diagnostic accuracy highlight its significance in both clinical practice and research settings [29,30,31].

**Figure 5:**
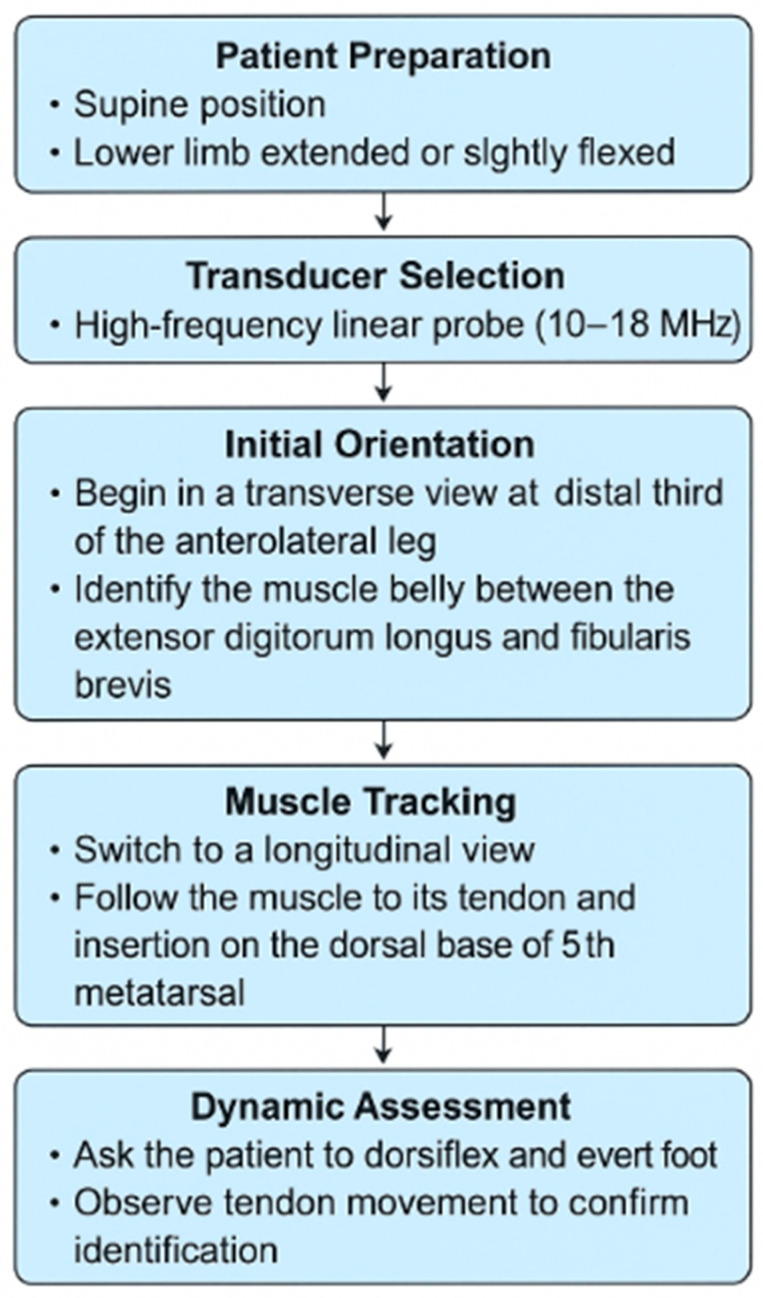
Ultrasonographic evaluation algorithm for the fibularis tertius muscle.

**Figure 6:**
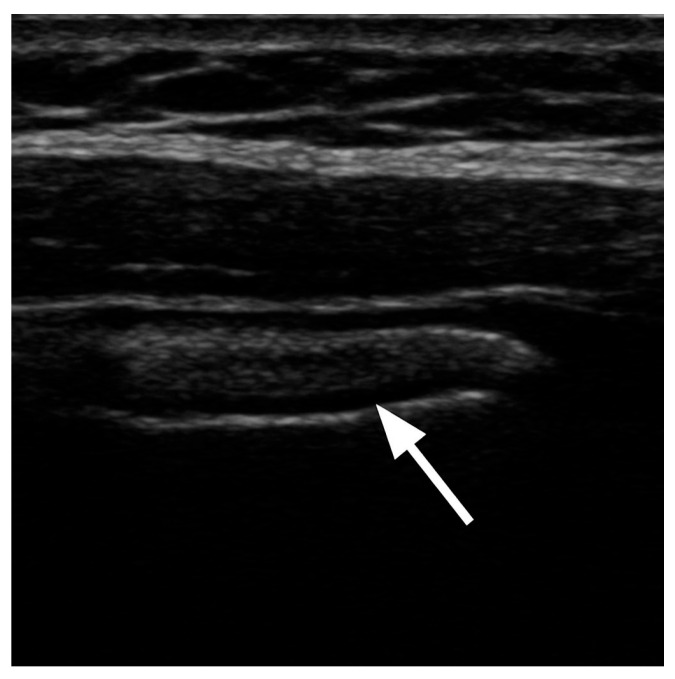
Ultrasound image of the fibularis tertius muscle in a lateral view, with the patient lying in a supine position. The muscle is marked with an arrow.

High-resolution ultrasonography (HR-US) is a valuable modality for identifying muscular variants, including the FT, and for assessing its structure and function in real time. It allows for precise evaluation of the cross-sectional area, thickness and echotexture, facilitating the differentiation of muscle hypertrophy, atrophy or post-traumatic changes [32,33]. Owing to these capabilities, HR-US plays a crucial role in diagnosing compression syndromes, chronic ankle instability, tendon subluxation and is instrumental in preoperative planning [2,32,33]. Figure 5 presents a schematic of the procedure for visualizing the FT, while Figure 6 shows the FT as visualized during US imaging.

## 6. Clinical Significance—Pathological Background

### 6.1. Fibularis Tertius or Peroneus Tertius Syndrome (FTS or PTS)

FTS or PTS was introduced and defined by Iceman et al. [29] as a symptomatic form of FTT that causes catching or locking in the anterolateral ankle or rearfoot, accompanied by pain [29]. They believed that PTS may be secondary to impingement and constriction of the FTT as it passes beneath the inferior extensor retinaculum (ER). The authors conducted a retrospective case series involving four patients diagnosed with PTS and described an operative technique for resecting the symptomatic tendon. The diagnosis of PTS was made clinically based on the patients’ symptoms, which included catching or locking of the FTT over the anterolateral ankle or rearfoot, accompanied by pain [29]. All patients underwent non-contrast MRI and shared typical findings—increased signal intensity surrounding the FTT on T2-weighted images and a low-lying muscle belly [29]. All surgeries were performed in the supine position, under general anaesthesia, with the use of a thigh tourniquet. The method assumes execution of two incisions—a 3 cm incision overlaying the dorsolateral aspect of the fifth metatarsal bone and a 2 cm incision proximal to the anterolateral aspect of the ankle joint overlying the FT’s musculotendinous junction, and extraction of the entire tendon and a portion of the FT muscle belly at the most proximal aspect [29]. The follow-up ranged from 8.5 to 27 months. At the final follow-up, patients were given a questionnaire to evaluate their postoperative outcomes and satisfaction (on a scale from 1 to 5; 1—very dissatisfied; 5—very satisfied), rate functional status (on a scale from 1 to 3; 1—decreased; 3—improved) and rate overall pain on the VAS scale. All patients demonstrated complete resolution of their symptoms and reported improved functional status with no evidence of recurrent symptoms during the final follow-up [29].

According to Iceman et al. [29] the diagnosis of PTS may be challenging to establish due to the patient’s vague anterolateral ankle or rearfoot pain. The authors recommend obtaining an MRI to confirm the presence of an FT and rule out other aetiologies of anterolateral ankle pain (such as proliferative synovitis, ligamentous injury, soft tissue impingement, osteochondral lesions, loose bodies and arthritis before surgical intervention). MRI is also functional for preoperative planning because of the variable muscle belly and tendon anatomy [29].

### 6.2. Fibularis Tendon Tertius (FTT) Tear

FTT injury is an infrequent cause of ankle pain. To our knowledge, there are only three reports of FTT tear in current literature. Barchi et al. [34] reported a case of a complete FTT tear in a 24-year-old female professional ballet dancer. She experienced acute pain in her right lateral foot after hyperflexing while wearing her pointe shoe. The dancer underwent an entire course of physical therapy and took non-steroidal anti-inflammatory drugs. Despite this treatment, her pain and swelling in the lateral ankle worsened when she returned to dancing. An MRI was performed and confirmed a complete FTT tear with retraction. The patient then underwent surgical debridement of the FTT and successfully returned to “en pointe” dancing within 9 weeks [34]. Derrick et al. [30] reported a case of a 39-year-old female who presented with pain, burning sensation and swelling of the left ankle with suspicion of the anterior talofibular ligament tear and an osteochondral defect. MRI examination was commissioned and revealed that the patient had both FT and fibularis quartus (FQ), a high-grade tendon tear injury of the FTT and a complex longitudinal split tear of the fibularis brevis tendon (FBT) [30]. The case report did not include the treatment method used for the patient, apart from the diagnostic images.

McGoldrick et al. [31] presented an intriguing case of a full-thickness tear of the FTT in a 12-year-old child who experienced two injuries in quick succession. Three months before their visit, the patient suffered a twisting injury. However, since the symptoms resolved quickly, he did not seek treatment at that time. The symptoms re-emerged after a second injury, which involved forceful plantarflexion and inversion. This incident resulted in sudden, sharp anterolateral ankle pain, bruising and swelling. Upon examination, his findings were consistent with a grade 2 sprain of the lateral ligament complex. The patient was fitted with an air cast boot for comfort and began physical therapy. Four weeks after the injury, the swelling in his ankle had subsided, and his range of motion in the tibiotalar joint was normal. Despite this improvement, he remained hesitant to bear weight, and tenderness persisted in the anterolateral ankle joint. An MRI scan indicated possible FTT pathology, leading to a decision for surgical exploration. A complete, full-thickness tear of the FTT was confirmed during the surgery. The tendon was repaired proximally using a suture. At the final follow-up visit, nine months post-operation, the patient’s pain had resolved, and he was able to bear full weight on the affected ankle. The findings from previously published literature highlight the importance of considering an FTT tear during the assessment and evaluation of lateral ankle pain [34].

### 6.3. Intra-Tendinous Ganglion Cyst

Ganglion cysts are thought to develop from the myxoid degeneration of connective tissue, resulting in a gelatinous substance within a cyst-like capsule. Although the foot and ankle are the second most common locations for ganglion cysts—after the hand and wrist—this area accounts for only 3–5% of all cases [35,36,37]. Intra-tendinous ganglion cysts are uncommon in both the upper and lower limbs. However, Walls et al. [37] reported an interesting case involving an intra-tendinous ganglion cyst of the fibular tendon. The authors presented the case of a 58-year-old male who experienced right foot pain for three years due to a mass on the midfoot’s dorsolateral aspect. An MRI revealed a ganglion cyst originating from the FTT sheath. The lesion was successfully decompressed, but it recurred seven months later. Since the cyst was causing symptoms, the authors opted for surgical resection. During the surgery, they found that the cyst had developed from an intrasubstance tear of the peroneus tertius tendon, and a branch of the superficial peroneal nerve was adherent to the pseudo-capsule. After the lesion and its surrounding pseudo-capsule were excised, the tendon tear was repaired through a tubularization technique, followed by external neurolysis of the affected nerve. Six months after the surgery, the patient was symptom-free, with no recurrence of the lesion and a complete restoration of normal physical function [37].

## 7. Clinical Application

### 7.1. Ankle Arthroscopy and FTT

According to Ilyas et al. [38], the FTT is a reference point for the anterolateral portal during ankle arthroscopy. This portal is used to place the inflow cannula and is established under direct visualization. It is typically created just lateral to the FTT or immediately above the joint line level [38]. This portal is not commonly used due to its proximity to the anterior neurovascular bundle [39]. The branches of the superficial peroneal nerve are at high risk. However, it offers certain advantages, such as a wide field of vision, rendering portal changes unnecessary. Stotter et al. [39] conducted a study assessing neurovascular complications following anterior ankle arthroscopy via the anteroventral portal. The overall complication rate was 7.6%, and no significant complications were reported.

### 7.2. Local Muscle Flap Transposition

Arnold et al. [40] published an intriguing article about muscle flaps in treating osteomyelitis. Osteomyelitis is a destructive disease of bone tissue caused by infection with pathogenic microorganisms. Due to its complex and prolonged nature, it is considered one of the more challenging diseases to treat within orthopaedics [41]. One approach to treating chronic osteomyelitis involves managing the dead space resulting from aggressive marrow cavity debridement. To fill the void created by this debridement, many surgeons recommend using various muscle flaps due to their convenience and anti-infective properties [42]. In a study conducted by Arnold et al. [40], the authors examined 64 patients who underwent local muscle flap transposition as a crucial part of their treatment for lower-extremity osteomyelitis. The muscles involved in the transposition included the medial gastrocnemius, soleus, gastrocnemius and, in one case, the FT. At the final follow-up, the recurrence-free rates at 5, 10 and 15 years were recorded at 94%, 92.5% and 86%, respectively. These long-term results underscore the effectiveness of local muscle flap transposition as a vital strategy in managing lower-extremity osteomyelitis [40].

### 7.3. Equinovarus Deformity

Most equinovarus foot deformities are caused by neurological conditions, with Charcot–Marie–Tooth disease being the most common. This deformity results from an imbalance of muscles that causes the foot and ankle to rotate into equinus (plantar flexion) and varus (inward) positions. The surgical approach to treating an equinovarus foot involves several key steps: releasing contracted soft tissues, assessing the bone deformity and correcting it by adjusting the calcaneus (heel bone) and metatarsal bones. Muscle rebalancing is essential after tendon transfer to ensure the correction is maintained. For tendon transfers, options include partial or complete transfer of the anterior tibial tendon, posterior tibial tendon or peroneal tendon. Typically, the transferred tendon is anchored to either the middle or lateral cuneiform bones or the cuboid bone [43,44,45]. Hoffer et al. [44] presented tibial tendon transfer to the foot to treat recurrent clubfoot and suggested that these tendons could also be transferred to address other severe structural deformities. Dong et al. [45] reviewed the results associated with a tendon transfer technique following the release and correction of talipes equinovarus, focusing mainly on the complications of this method. Their study included 176 patients (210 feet) who underwent the procedure. The researchers assessed both passive and active range of motion in the foot and ankle before and after the surgery. Postoperative radiographic evaluations comprised anteroposterior (AP), lateral and hindfoot alignment views. Specific measurements taken preoperatively and postoperatively included the lateral tibiotalar angle, talo-calcaneal angle, talo-first metatarsal angle, tibial-sole angle, hindfoot alignment and the anterior subluxation of the talus. For pain assessment, they utilized the American Orthopaedic Foot and Ankle Society (AOFAS) ankle–hindfoot scale and the Visual Analog Scale (VAS) [43]. Researchers observed significant differences between preoperative and postoperative measurements, AOFAS and VAS scores and established that anterior/posterior tibial tendon transfer to the foot and ankle is a safe and effective method for correcting equinovarus deformity [45].

### 7.4. Lateral Ankle Stabilization

Ankle sprains are common injuries that involve the lateral ankle ligaments. Most patients heal without complications, but if symptoms persist three months after the injury, an evaluation for chronic ankle instability (CAI) is necessary. CAI is initially treated with non-operative methods; however, if functional rehabilitation fails after three to six months, surgical options should be considered. The preferred initial surgery is an anatomic repair using a modified Brostrom procedure. Anatomic reconstruction with a tendon graft should only be considered when repair is impossible. The FBT is the most frequently used material for grafting [46] Sammarco et al. [47] presented an intriguing study involving 10 patients who required revision ankle reconstruction after their initial lateral ankle stabilization surgery failed. In seven of the ten patients, the revision ligament reconstruction utilized tendon grafts, including the FB, plantaris, accessory peroneus and FT. The surgical procedures for the revision reconstruction involved the modified Elmslie technique, which was performed using a split peroneus brevis tendon in four patients. In the remaining three patients, similar reconstructions were conducted using different tendons: one patient received a graft from the plantaris tendon, another from the FTT and a third from the AFT. The FBT, used for the primary reconstruction, was not available for these revision surgeries. The authors concluded that 9 out of the 10 patients experienced good or excellent results, and all 10 patients achieved stable ankles following the procedure [47].

## 8. Conclusions

FT is a fascinating structure that has captured the attention of scientists in various fields, including medical, clinical and biological sciences, for years. This comprehensive literature review highlights FT’s morphology, anatomical variability, function, evolution and clinical significance, based on the current scientific understanding of this structure.

## Figures and Tables

**Table 1:** Comparison of prevalence of the fibularis tertius (FT) in adults between different studies (based on Yammine and Erić., 2017 [7]), according to the year of publication.

Author(s)	Year	Ethnicity	Study Type	Prevalence (%)
Unknown	-	Bahrain	Cadaver	42.0
Unknown	-	Saudi	Cadaver	38.5
Unknown	-	Kuwaiti	Cadaver	41.2
Wood	1866	British	Cadaver	95.3
Le Double	1897	French	Cadaver	94.2
Koganei et al.	1903	Japanese	Cadaver	96.7
Adachi	1909	Japanese	Cadaver	95.0
Loth	1913	African	Cadaver	90.2
Nakano	1923	Chinese	Cadaver	89.3
Posmykiewicz	1934	Polish	Clinical	92.6
Werneck	1957	Caucasian, Black	Cadaver	95.6
Sokolowska-Pituchowa et al.	1974	Polish	Cadaver	92.0
Krammer et al.	1979	Austrian	Cadaver	92.9
Testut, Latarjet	1979	Israeli	Cadaver	10.0
Reimann	1981	German	Cadaver	90.0
Bertelli and Khoury	1991	French	Cadaver	91.0
Stevens et al.	1993	British	Cadaver	95.0
Da-Yae Choi1	2001	Korean	Cadaver	92.6
Larico and Jordan	2005	Bolivian	Cadaver	100.0
Joshi et al.	2006	Indian	Cadaver	89.6
Kunnika et al.	2006	Thai	Cadaver	95.6
Marin et al.	2006	Brazilian	Cadaver	94.0
Marin et al.	2006	Brazilian	Cadaver	93.8
Witvrouw et al.	2006	Belgian	Clinical	81.5
Rourke et al.	2007	British	Cadaver	93.9
Bhatt et al.	2010	Indian	Cadaver	89.4
Ramirez et al.	2010	Chilean	Clinical	50.9
Bourdon and Petitdant	2012	French	Clinical	88.4
Ashaolu et al.	2013	Nigerian	Clinical	63.0
de Gusmão et al.	2013	Brazilian	Cadaver	96.9
Oyedun et al.	2014	Nigerian	Clinical	88.5
Surekha et al.	2015	Indian	Cadaver	87.0
Verma and Seema	2015	Indian	Cadaver	100.0
Ercikti et al.	2016	Turkish	Cadaver	95.5
Losa-Iglesias et al.	2017	Spanish	Clinical	38.2
Salem et al.	2018	Tunisian	Clinical	67.7
Salem et al.	2018	Egyptian	Clinical	52.8

**Table 2:** A comparison between sites of origin of fibularis tertius (FT) among different authors (based on Yammine and Erić); EDL: extensor digitorum longus.

Author	Year	Origin [%]		
		Distal Half Fibula	Distal Third Fibula	EDL Tendon
Kaneff	1980	75.7	0	24.3
Bertelli and Khoury	1991	100	0	0
Stevens et al.	1993	92.11	-	-
Marin et al.	2006	83.3	16.7	0
Rourke et al.	2007	100	0	0
Bhatt et al.	2010	92.8	0	7.14
de Gusmão et al.	2013	45.2	54.8	0
Surekha et al.	2015	91.9	0	8.04
Verma and Seema	2015	98.3	0	1.66
Olewnik	2019	67	22	11

**Table 3:** A comparison between insertion sites of fibularis tertius (FT) among different authors (based on Yammine and Erić [7]), according to the year; M5: 5th metatarsal bone; M4: 4th metatarsal bone.

Author(s)	Year	Insertion Sites %				
		Shaft M5	Base M5	M4–M5	M4	EDL Tendon
Wood	1866	90.2		3.28	6.5	0
Wood	1867	93.3			6.7	0
Wood	1868	88.4		2.90	4.3	4.3
Johnson	1973	10.9	47.3	12.4	1.2	16
Kaneff	1980	14	75.7	10	0	0
Bertelli and Khoury	1991	10	80	0	0	10
Stevens et al.	1993	82.5	-	-	-	10
Domagala et al.	2006	9.9	0	90	0	0
Marin et al.	2006	90	7	3	0	0
Rourke et al.	2007	0	0	100	0	0
de Gusmão et al.	2013	77.4	0	22.6	0	0
Surekha et al.	2015	0	44.8	25.3	24.1	0
Verma and Seema	2015	100		0	0	0
Olewnik	2019	45	22	0	0	0

**Table 4:** The fibularis tertius (FT) presence in different studies among foetuses based on Yammine and Erić [7].

Author(s)	Year	Ethnicity	Study Type	Fetuses	Prevalence
Sokolowska-Pituchowa et al.	1979	Polish	Cadaver	Fetuses	78.6%
Kaneff	1980	French	Cadaver	Fetuses	88.2%
Domagala et al.	2006	Polish	Cadaver	Fetuses	83.2%
Albay and Candan	2017	Turkish	Cadaver	Fetuses	80.0%
Karauda et al.	2021	Polish	Cadaver	Fetuses	50.0%
Ruzik et al.	2022	Polish	Cadaver	Fetuses	50.0%
